# Significant changes in soil microbial community structure and metabolic function after *Mikania micrantha* invasion

**DOI:** 10.1038/s41598-023-27851-6

**Published:** 2023-01-20

**Authors:** Panpan Zhao, Biying Liu, Hengjun Zhao, Zhengyan Lei, Ting Zhou

**Affiliations:** State Key Laboratory of Biocontrol, School of Life Sciences, Sun Yat-sen University, Guangzhou, 510275 China

**Keywords:** Ecology, Microbiology, Plant sciences

## Abstract

Currently, *Mikania micrantha* (*M. micrantha*) has invaded Guangdong, Guangxi and other provinces in China, causing serious harm to the forests of southeastern China. Soil microorganisms play an important role in the establishment of *M. micrantha* invasion, affecting plant productivity, community dynamics, and ecosystem function. However, at present, how *M. micrantha* invasion affects soil carbon, nitrogen, and phosphorus phase functional genes and the environmental factors that cause gene expression changes remain unclear, especially in subtropical forest ecosystems. This study was conducted in Xiangtoushan National Forest Park in Guangdong Province to compare the changes in soil nutrients and microorganisms after *M. micrantha* invasion of a forest. The microbial community composition and metabolic function were explored by metagenome sequencing. Our results showed that after *M. micrantha* invasion, the soil was more suitable for the growth of gram-positive bacteria (Gemmatimonadetes). In addition, the soil microbial community structure and enzyme activity increased significantly after *M. micrantha* invasion. Correlation analysis and Mantel test results suggested that total phosphorus (TP), nitrate nitrogen (NO_3_^–^-N), and soil dissolved organic matter (DOM; DOC and DON), were the strong correlates of soil microbial nitrogen functional genes, while soil organic matter (SOM), soil organic carbon (SOC), total nitrogen (TN), and available phosphorus (Soil-AP) were strongly correlated with the expression of soil microbial phosphorus functional gene. *Mikania micrantha* invasion alters soil nutrients, microbial community composition and metabolic function in subtropical forests, creates a more favorable growth environment, and may form a positive feedback process conducive to *M. micrantha* invasion.

## Introduction

Invasive species are considered to be the second largest factor leading to the decline in global biodiversity^1^. Alien plant invasion occurs in various ecosystems, which is an important aspect of global change and has a significant negative impact on ecosystem community structure and function, diversity and productivity^2,3^. For example, invasive plants can reduce the local plant species diversity^4–7^, increase the aboveground biomass and net primary productivity of the ecosystem and change the nutrient cycle of soil carbon, nitrogen and phosphorus^8,9^. It is well known that the link between plants and soil microorganisms is essential^10^. Microorganisms are very important in regulating plant productivity, community dynamics and soil formation^11^. Studies have shown that soil microorganisms play an important role in the establishment of plant invasion and may also be the driving factors of plant invasion^12^.

Recently, researchers have suggested that soil microorganisms play an important role in plant competitiveness through negative plant‒soil feedbacks^13^ and allelopathy^14^. After plant invasion, soil microorganisms improve the nutrient supply of soil, thus promoting plant invasion. The results showed that the soil microbial community changed significantly after the invasion of exotic plants^15^. For example, after the invasion of *M. micrantha*, the microbial biomass, soil respiration, microbial carbon utilization efficiency, microbial community structure (phospholipid fatty acid analysis, PLFA)^16,17^ and bacterial diversity were significantly different^18^. Recent studies have also shown that there are significant differences in microbial community structure between invasive species and native species (*Polygonum chinense* and *Paederia scandens*) of *M. micrantha* in pot and field invasion environments^19^. The change in microbial community structure is not necessarily accompanied by a significant change in community functional diversity^20,21^. Some studies have noted that after *M. micrantha* invasion, the change in function (community-level physiological profiles, CLPP) of the microbial community is greater than that of its structure (PLFA), and the response of function is more sensitive^17^, but research on metabolic function after invasion is not sufficiently comprehensive.

Invasive plants always have a stronger ability to capture resources^22^ and to take advantage of infrequent pulses in resource availability^23,24^. Studies have shown that one of the reasons for the success of invasive plants is the increase in nitrogen availability and nitrogen transfer rates due to changes in the rates of atmospheric nitrogen fixation and plant nitrogen uptake in the soil after invasion, which can also improve the competitiveness of plants^25^. Previous studies have shown that invasive plants increase the conversion rates and availability of N and P in ecosystems^25–32^, as well as carbon storage^9,33–35^. Yu et al.^36^ studied how the soil nitrogen cycle was affected by microorganisms mediated after growth of the invasive plant *M. micrantha* and two native plants, the *Chinese Persian flower* and *Uncaria officinalis*. The results showed that the *M. micrantha* rhizosphere had highly diverse nitrogen cycle microorganisms, including nitrogen fixation bacteria, ammonia oxidation bacteria (AOB), denitrification bacteria and ammonia oxidation archaea (AOA). *Mikania micrantha* mainly obtains available nitrogen through AOA-mediated nitrification. Previous studies were limited to the description of microbial community composition and abundance, as well as the changes in nitrogen-related functional genes. The cycling and availability of soil carbon, nitrogen and phosphorus nutrients are closely related not only to the soil microbial community^32^ but also to their respective functional genes. The changes in soil carbon and phosphorus mediated by soil microbial function may also be an important reason for the invasion of *M. micrantha*, but little attention has been given to the changes in carbon and phosphorus-related functions at the genetic level.

*M. micrantha*, a perennial vine in the Asteraceae family, is native to tropical America^37^ and is one of the top 100 worst invasive species identified by the International Union for Conservation of Nature (IUCN)^38^. *Mikania micrantha* invades tropical Asia, parts of Papua New Guinea, regions in the Indian Ocean and Pacific Ocean islands, and Florida in the US^39,40^. *Mikania micrantha* appeared as a weed in Hong Kong, China, in 1919 and has now spread to Guangdong, Guangxi, Fujian, Hainan, Yunnan and other provinces^39^, causing serious substantial damage to the natural ecosystem^37^ and economic losses^41^. In this study, samples were taken from two forests (a pristine forest and a forest invaded by *M. micrantha*) in Xiangtoushan National Forest Reserve in Guangdong Province. The microbial mechanism of *M. micrantha* invasion of forests was explored by studying the changes in soil microbial species composition and soil microbial metabolic function after *M. micrantha* invasion and their relationship with environmental factors. We measured the differences in soil temperature and moisture (SWC); soil organic matter (SOM), soil organic carbon (SOC), nitrogen (TN) and phosphorus (TP) and available nutrients; dissolved organic matter (DOC, DON); soil texture; and microbial biomass, composition, diversity and functional metabolism between the two kinds of forests. The aims of this study were to address the following questions: (1) What are the effects of *M. micrantha* invasion on the soil microbial community composition, diversity and metabolic function? We would like to know if there are differences in microbial species or metabolism that improve the competitiveness of *M. micrantha* to native plants. (2) How does soil microbial composition and functional gene expression of carbon, nitrogen and phosphorus cycling change after *M. micrantha* invasion? How do they relate to environmental factors? We hypothesized that (1) after *M. micrantha* invasion, the microbial community composition and metabolic function beta diversity changed significantly, and some nutrient-related composition and metabolic genes more conducive to the growth of invasive plants will change, such as Gemmatimonadetes; and (2) after *M. micrantha* invasion, and the functional gene expression and enzyme activities related to soil nitrogen and phosphorus may increase. Soil nitrate nitrogen (NO_3_^–^-N), available phosphorus (soil-AP), and DOM (DOC and DON) were significantly correlated with microbial species composition and functional genes. The soil physical and chemical environment and microorganisms provide a more favorable growth environment for *M. micrantha*.

## Materials and methods

### Site description

Our study was conducted at the Xiangtoushan National Nature Reserve (23°13′05ʺ ~ 23°19′43ʺN, 114°19′21ʺ ~ 114°27′06″E) located in Guangdong Province, China. The reserve has a humid monsoon climate of the southern subtropics, with sufficient light, abundant heat, abundant precipitation, humid air, a short dry season, a long wet season, a long plant growth period and a long frost-free period, and directional wind changes with the seasons. The annual average temperature is 16.0–22.8 °C in this area. January or February are the coldest months, with monthly average temperatures of 7.2–13.3 °C; August the hottest month with average temperatures of 22.5–27.2 °C. The annual precipitation is 2318.5 mm. From April to September, the precipitation is the highest, and the guaranteed rate of the annual precipitation of 1601–2000 mm is 94%. It varies greatly from year to year, with a maximum of 3516 mm and a minimum of 1012 mm. The highest elevation of Xiangtou Mountain is 1024 m, where the annual average air relative humidity is 80%, and the forest coverage is 88.4%. The hydrothermal conditions and geographical location of Xiangtoushan National Nature Reserve provide natural environmental conditions for the invasion and reproduction of *M. micrantha*.

**Figure 1.**
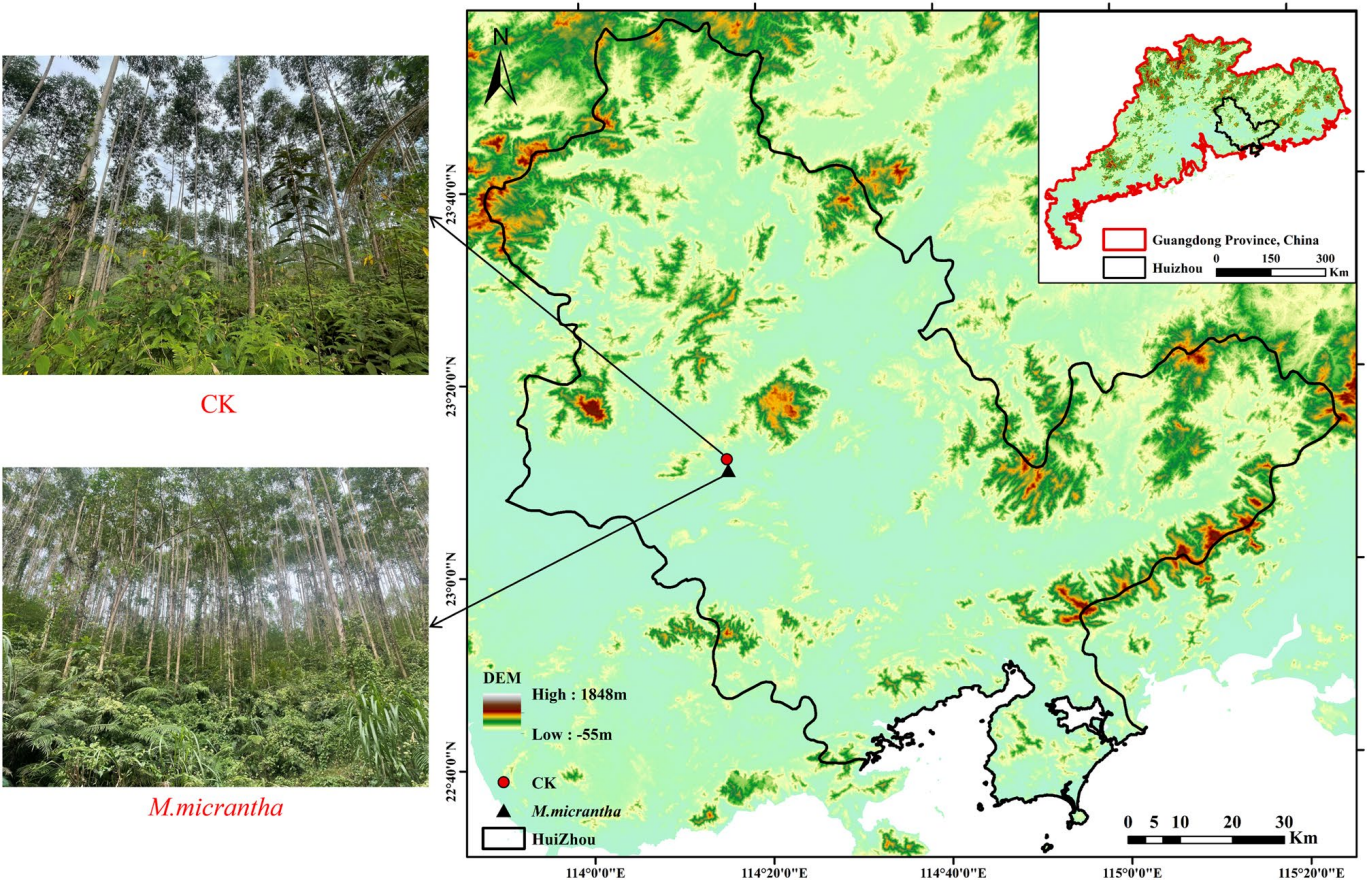
Spatial distribution of sampling sites in Xiangtoushan National Nature Reserve, Guangdong province, China. The black solid triangle represents *M. micrantha* site while the red solid circle represents CK site. In addition, the depth of color represents the elevation in the DEM. And the map was generated with ArcGIS 10.2 (ESRI, Inc., Redlands, CA, USA, http://www.esri.com/software/arcgis/arcgis-for-desktop).

In our study, we selected two kinds of forests with the same site conditions, the pristine forest (control, CK) and *M. micrantha*-invaded forest (M.I). These forests had the same dominant tree species (*Eucalyptus robusta*, *E. robusta*), and the dominant species in the understory shrub layer was *Dicranopteris dichotoma*, which was accompanied by *Clerodendrum fortunatum*, *Schefflera heptaphylla*, *Mussaenda pubescens*, and *Blechnum orientale*. The soils of the sample plots are all lateritic red soils developed from granite. The main characteristics of the studied sites are summarized in Fig. 1.

### Soil sampling

In December 2020, we selected pristine forest and *M. micrantha*-invaded forest of Xiangtou Mountain for sampling. The invasion time of *M. micrantha* is approximately 30 years, and the coverage of *M. micrantha* invasion is 60%. In the forest invaded by *M. micrantha*, soil was sampled 50 cm away from *E. robusta*. Three 20 m × 20 m standard sampling plots were set up under each stand, and 5 m × 5 m sampling areas were randomly arranged in each standard sample plot. Each small sample area was sampled by the "S" mixed sampling method. After the litter was removed, five topsoil cores (5 cm diameter and 10 cm in depth) were collected from random spots in each plot and combined and homogenized. At each location, three sampling plots were separated by more than 100 m. In total, 6 samples (2 treatments × 3 replications) were placed in sealed plastic bags and then immediately transported to the laboratory. The stones and roots were removed, and each fresh soil sample was ground to pass through a 2-mm mesh sieve and then divided into two subsamples. One subsample of soil was prepared to determine soil ammonium (NH_4_^+^-N), NO_3_^–^-N, soil-AP, microbial biomass carbon (MBC), microbial biomass nitrogen (MBN), enzyme activities and DNA extraction. The other subsample of air-dried soil was passed through a 0.149-mm sieve to analyze the other soil properties.

### Measurements of soil properties

The soil temperature was determined using a handheld thermometer (SK-250 WP, SK Stoto, Japan). The SWC was determined by oven drying for 48 h at 105 °C and calculated using the wet and dry weights. Soil pH was measured using a pH meter, in a 1:2.5 (w/v) soil‒water suspension. The SOM and SOC contents were determined by the high-temperature external heat potassium dichromate oxidation volumetric method. The TN content was measured using an elemental analyzer (Elementar Vario EL III; Elementar, Germany). To determine the TP content, samples were digested with 4:1 H_2_SO_4_/HClO_4_^42^, and then the supernatant was passed through a 0.45-μm glass fiber filter (Q/IEF J01-1997, Shanghai) and analyzed using a continuous-flow analytical system (Skalar San++; Skalar, the Netherlands). A 3-g air-dried soil sample was mixed with 30 mL of Mehlich-3 extracting solution, shaken immediately for 5 min, and centrifuged for 5 min at 8000 × *g*. The supernatant was used to determine the soil-AP content. NH_4_^+^-N and NO_3_^–^-N were extracted using 2 M KCl and measured using the continuous-flow analytical system^43^. Soil MBC and MBN contents were measured using the chloroform fumigation–extraction method^44^.

### Methods of DNA extraction, library preparation and metagenome sequencing

The samples were transported to the laboratory on ice and stored at − 80 °C until DNA was extracted from approximately 0.25 g of soil using the Magen Hipure Soil DNA Kit (MoBio Laboratories, Carlsbad, CA, USA). DNA concentrations were measured with a Qubit 2.0 fluorometer (Invitrogen, USA)^45^.

Next-generation sequencing library preparations were constructed following the manufacturer’s protocol (VAHTS Universal DNA Library Prep Kit for Illumina). For each sample, 200 ng of genomic DNA was randomly fragmented to < 500 bp by sonication (Covaris S220). The fragments were treated with End Prep Enzyme Mix for end repair, 5′ phosphorylation and dA tailing in one reaction, followed by T-A ligation to add adaptors to both ends. Size selection of adaptor-ligated DNA was then performed using VAHTSTM DNA clean beads, and fragments of ~ 470 bp (with an approximate insert size of 350 bp) were recovered. Each sample was then amplified by PCR for 8 cycles using P5 and P7 primers, with both primers carrying sequences that can anneal with flow cells to perform bridge PCR and P7 primers carrying a six-base index to allow for multiplexing. The PCR products were cleaned up using VAHTSTM DNA clean beads, validated using an Agilent 2100 bioanalyzer (Agilent Technologies, Palo Alto, CA, USA), and quantified by a Qubit 3.0 fluorometer (Invitrogen, Carlsbad, CA, USA).

Then, libraries with different indices were multiplexed and loaded on an Illumina HiSe instrument according to the manufacturer’s instructions (Illumina, San Diego, CA, USA). Sequencing was carried out using a 2 × 150 paired-end (PE) configuration; image analysis and base calling were conducted by HiSeq control software (HCS) + OLB + GAPipeline-1.6 (Illumina) on a HiSeq instrument.

Raw shotgun sequencing reads were trimmed using cutadapt (v1.9.1). Low-quality reads, N-rich reads and adapter-polluted reads were removed. Then, host contamination reads were removed using BWA (v0.7.12). Samples were each assembled de novo to obtain separate assemblies. Whole-genome de novo assemblies were performed using MEGAHIT (v1.13) with different k-mers. The best assembly result of Scaffold, which had the largest N50, was selected for the gene prediction analysis.

Genes of each sample were predicted using Prodigal (v3.02). CD-HIT was used to cluster genes derived from all samples with a default identity of 0.95 and coverage of 0.9. To analyze the relative abundance of unigenes in each sample, PE clean reads were mapped to unigenes using SOAPAligner (version 2.2.1) to generate read coverage information for unigenes. Gene abundance was calculated based on the number of aligned reads and normalized to gene length.

### Soil enzyme assay

Enzyme analysis was conducted following the procedure outlined by Saiya-Cork et al.^46^. We investigated two important soil carbon-related hydrolases and soil nitrogen and phosphorus-related hydrolases, including β-glucosidase (βG, EC 3.2.1.21), cellobiohydrolase (CBH, EC 3.2.1.91), N-acetyl-β-D-glucosaminidase (NAG, EC 3.2.1.30) and acid phosphatase (AP, EC 3.2.3.2). Briefly, 1 g of soil was homogenized in 125 mL of 50 mmol L^−1^ acetate buffer (pH 5.0), and then 200 µL of acetate buffer was added to a 96-well microplate. The microplate was incubated in the dark for 4 h at 20 °C. After incubation, 1 M NaOH (4 µL) was added to quench the reaction. The sample fluorescence was measured using 365-nm excitation and 450-nm emission filters on a SpectraMax M5 Microplate Reader (MDS Analytical Technologies, Molecular Devices, USA). All total enzyme activities were expressed as nmol g^−1^ soil h^−1^.

### Statistical analysis

Statistical analyses were performed with IBM SPSS Statistics 22 version 19.0 (SPSS Inc. Chicago, IL), STAMP (v2.1.3) and an R software package (version 4.1.0). Independent sample t tests were used to compare the differences in soil properties, soil microbial enzymes, soil microbial composition and soil microbial metabolic function between the CK and *M. micrantha*-invaded forests. Nonmetric multidimensional scaling (NMDS) was employed to analyze soil microbial community structure^19^ and microbial metabolic function based on the Bray‒Curtis distance in CK and *M. micrantha* invasion forests. We used Euclidean distances for composition (based on relative abundances at the phylum level), functional gene expression of carbon, nitrogen and phosphorus cycling and physical and chemical properties of soil and Haversine distances for geographic data. Given these distance matrices, we computed partial Mantel correlations between composition, functional gene and physical and chemical properties of soil given geographic distance (9999 permutations) using the *vegan* R software package.

## Results

### Soil properties

*M. micrantha* invasion significantly increased SWC, TP, NO_3_^–^-N, DON and MBN (*p* < 0.05), but significantly decreased DOC (*p* < 0.05). *Mikania micrantha* invasion had no effect on pH, SOM, SOC, TN, soil-AP, NH_4_^+^-N, MBC or soil texture (Table 1).

**Table 1:** Basic physicochemical characteristics of soils of the pristine forest forests and *M. micrantha*-invaded forest.

Treatment	Control	*M. micrantha*	*p*-value
SWC (%)	0.16 ± 0.01b	0.19 ± 0.07a	0.025
pH	4.88 ± 0.06a	4.99 ± 0.10a	0.181
SOM (g kg^−1^)	47.09 ± 7.34a	46.16 ± 9.49a	0.898
SOC (g kg^−1^)	27.32 ± 4.26a	26.77 ± 5.50a	0.899
TN (g kg^−1^)	2.03 ± 0.37a	2.10 ± 0.37a	0.981
TP (g kg^−1^)	0.22 ± 0.02b	0.33 ± 0.01a	0.001
AP (mg kg^−1^)	1.48 ± 0.44a	2.55 ± 0.57a	0.061
NH_4_^+^-N (mg kg^−1^)	12.25 ± 2.09a	15.19 ± 2.61a	0.203
NO_3_^–^-N (mg kg^−1^)	5.95 ± 0.37b	10.10 ± 2.56a	0.017
DOC (mg kg^−1^)	46.46 ± 2.17a	15.61 ± 1.99b	< 0.001
DON (mg kg^−1^)	34.17 ± 1.07b	48.38 ± 0.88a	< 0.001
MBC (mg kg^−1^)	45.63 ± 3.17a	39.45 ± 4.87a	0.145
MBN (mg kg^−1^)	9.04 ± 2.72b	22.20 ± 4.23a	0.011
Sand (2–0.05 mm, %)	45.37 ± 1.71b	39.00 ± 1.14a	0.023
Silt (0.05–0.002 mm, %)	37.73 ± 2.60a	42.00 ± 0.00a	0.115
Clay (< 0.002mmm, %)	16.90 ± 1.25a	19.00 ± 1.41a	0.175

Values are mean (n = 5). Different lowercase letters indicate significant differences at three altitudes in the same horizon. *SOM* Soil organic matter, *SOC* Soil organic carbon, *TN* Total nitrogen, *TP* Total phosphorus, *AP* Available phosphorus, *NH*_*4*_^*+*^*-N* Ammonium nitrogen, *NO*_*3*_^*−*^*-N* Nitrate nitrogen, *DOC* Dissolved organic carbon, *DON* Dissolved organic nitrogen, *MBC* Microbical biomass carbon, *MBN* Microbical biomass nitrogen.

### Soil microbial community composition and diversity.

**Figure 2:**
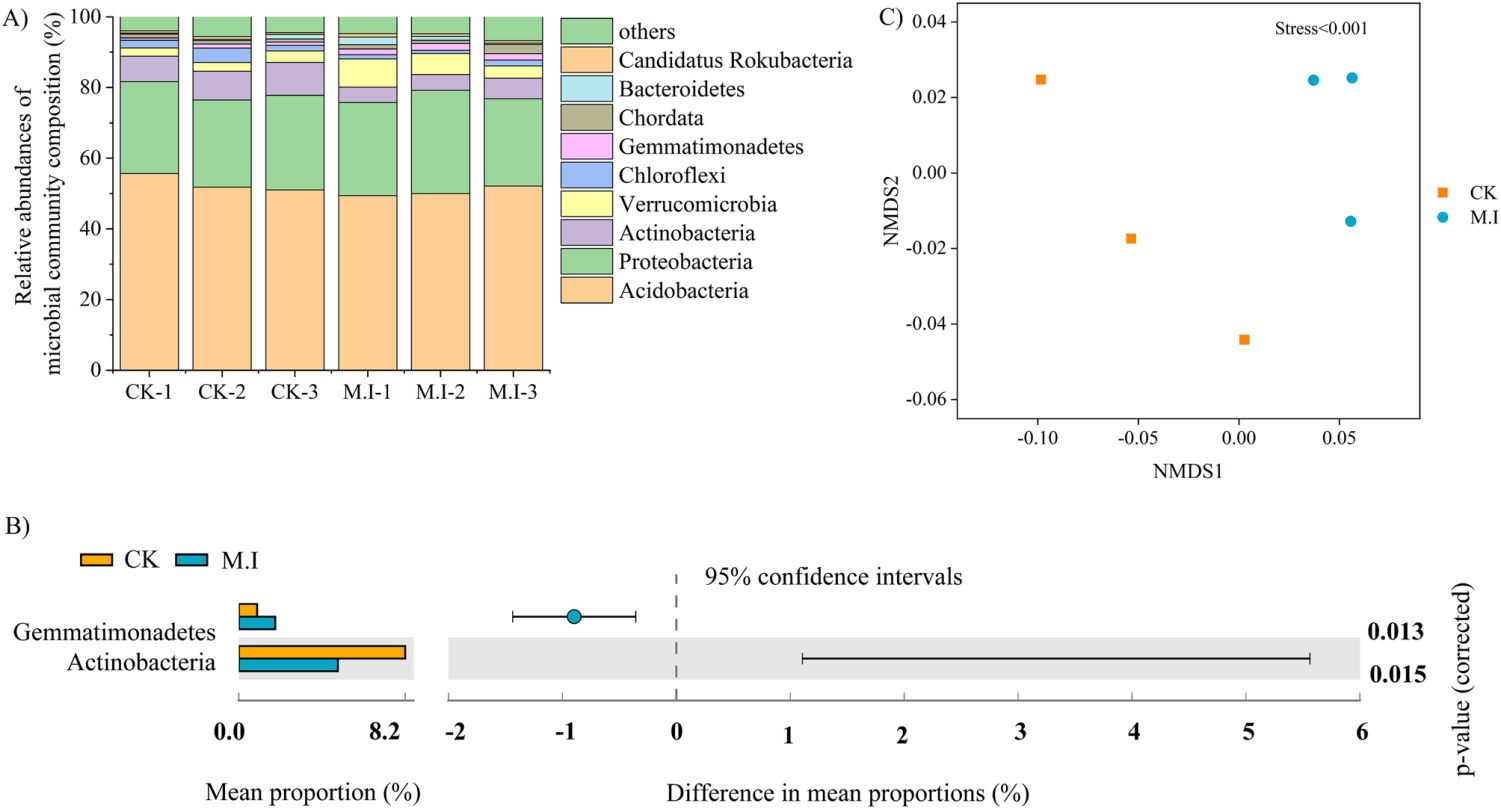
Microbial community composition and structure in soil of the pristine forest forests and *M. micrantha*-invaded forest. (**A**) Relative abundances at the phylum level in soil. (**B**) Differential species at the phylum level in soil. (**C**) The non-metric multidimensional scaling (NMDS) plot of microbial structures.

Metagenome sequencing resulted in approximately 17 million total sequences, averaging approximately 2,797,676 per sample. Basic information on microbial sequencing data statistics after past joint and low-quality treatment is shown in Table 2. In all forests, the microbial communities were predominantly composed of the phylum Acidobacteria (ranging from 49.44 to 59.66%), followed by Proteobacteria (ranging from 24.61 to 29.22%) (Fig. 2A). In addition, we analyzed the species at the genus level by pairwise difference comparison (Welch's t test), and the results showed that among the top 9 most abundant species, Gemmatimonadetes significantly increased after *M. micrantha* invasion (*p* < 0.05, Fig. 2B), but Actinobacteria significantly decreased (*p* < 0.05, Fig. 2B). Taxonomic changes indicated a shift from copiotrophic to oligotrophic groups after *M. micrantha* invasion.

**Table 2:** Sequencing data of macrogenes of the pristine forest forests and *M. micrantha*-invaded forest.

Sample	Total sequences (#)	Total bases (bp)	Average length (bp)	N50 (bp)	Length (nt)	Clean reads (#)	#Bases	Q20 (%)	Q30 (%)	GC (%)	N (ppm)
CK-1	2,737,132	1.90E+09	695.59	723	149.40	1.55E+08	2.31E+10	98.34	95.19	61.06	0.71
CK-2	2,621,812	1.77E+09	673.83	691	149.42	1.48E+08	2.21E+10	98.28	95.07	61.66	0.72
CK-3	2,776,257	1.97E+09	707.86	741	149.40	1.61E+08	2.40E+10	98.3	95.10	61.37	0.75
M.I-1	2,812,741	1.77E+09	630.28	634	149.41	1.47E+08	2.19E+10	98.33	95.22	61.20	0.67
M.I-2	3,152,549	2.02E+09	639.21	648	149.41	1.58E+08	2.37E+10	98.35	95.26	61.49	0.72
M.I-3	2,685,567	1.82E+09	676.10	699	149.43	1.52E+08	2.27E+10	98.31	95.17	61.36	0.72

Based on the species, we calculated the Bray‒Curtis distance between the sample pairs to understand the changes in microorganisms in the taxa and used NMDS to visualize this pattern (Fig. 2C). The NMDS plots showed that there was a clear separation between the CK forest and the forest invaded by *M. micrantha*, indicating that the soil microbial community was largely influenced by environmental conditions.

### Soil microbial metabolic function

**Figure 3:**
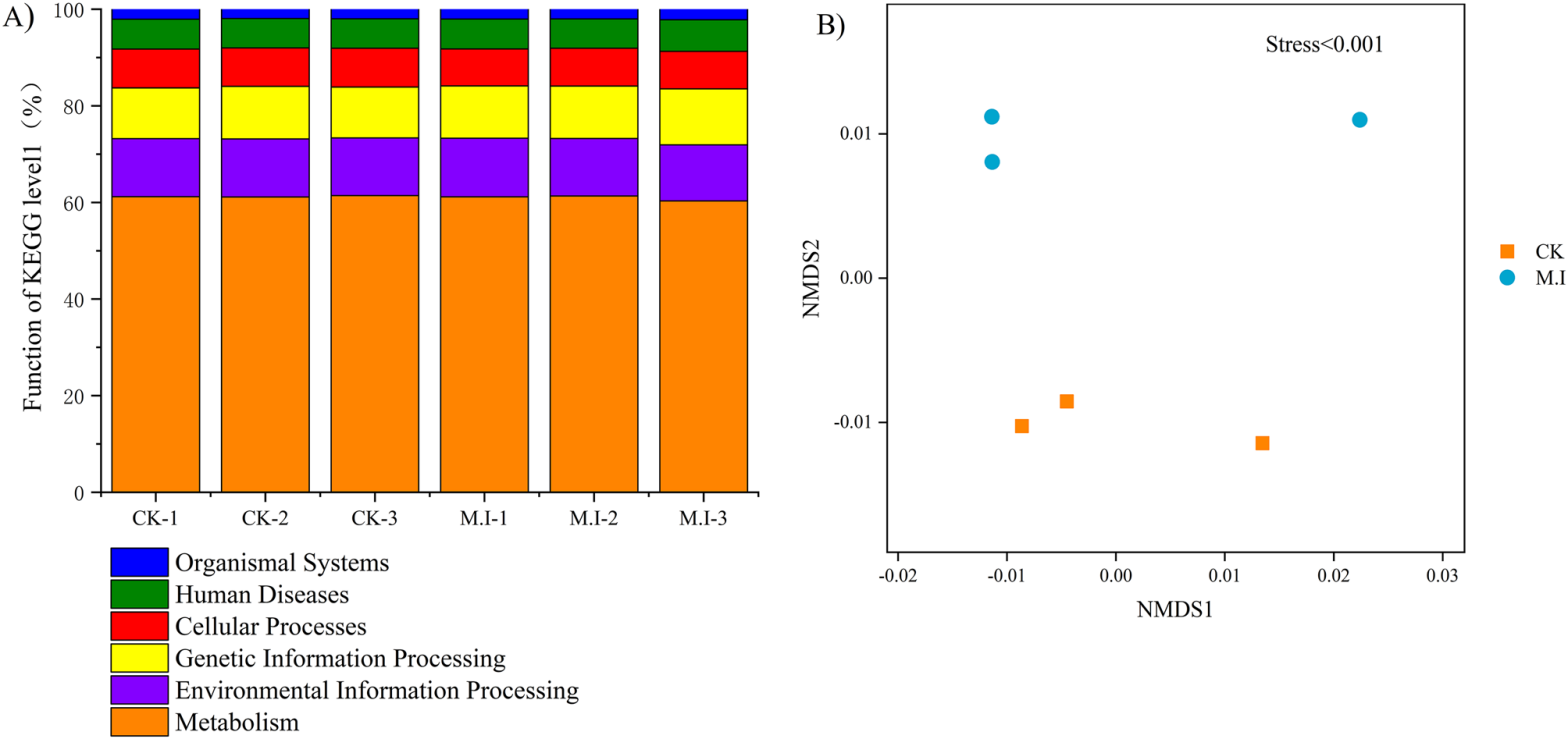
Microbial metabolic function in soil of the pristine forest forests and *M. micrantha*-invaded forest. (**A**) Relative abundances of soil microbial metabolic function at KEGG level 1. (**B**) The non-metric multidimensional scaling (NMDS) plot of microbial metabolic function at KEGG level 3 in soil.

**Figure 4:**
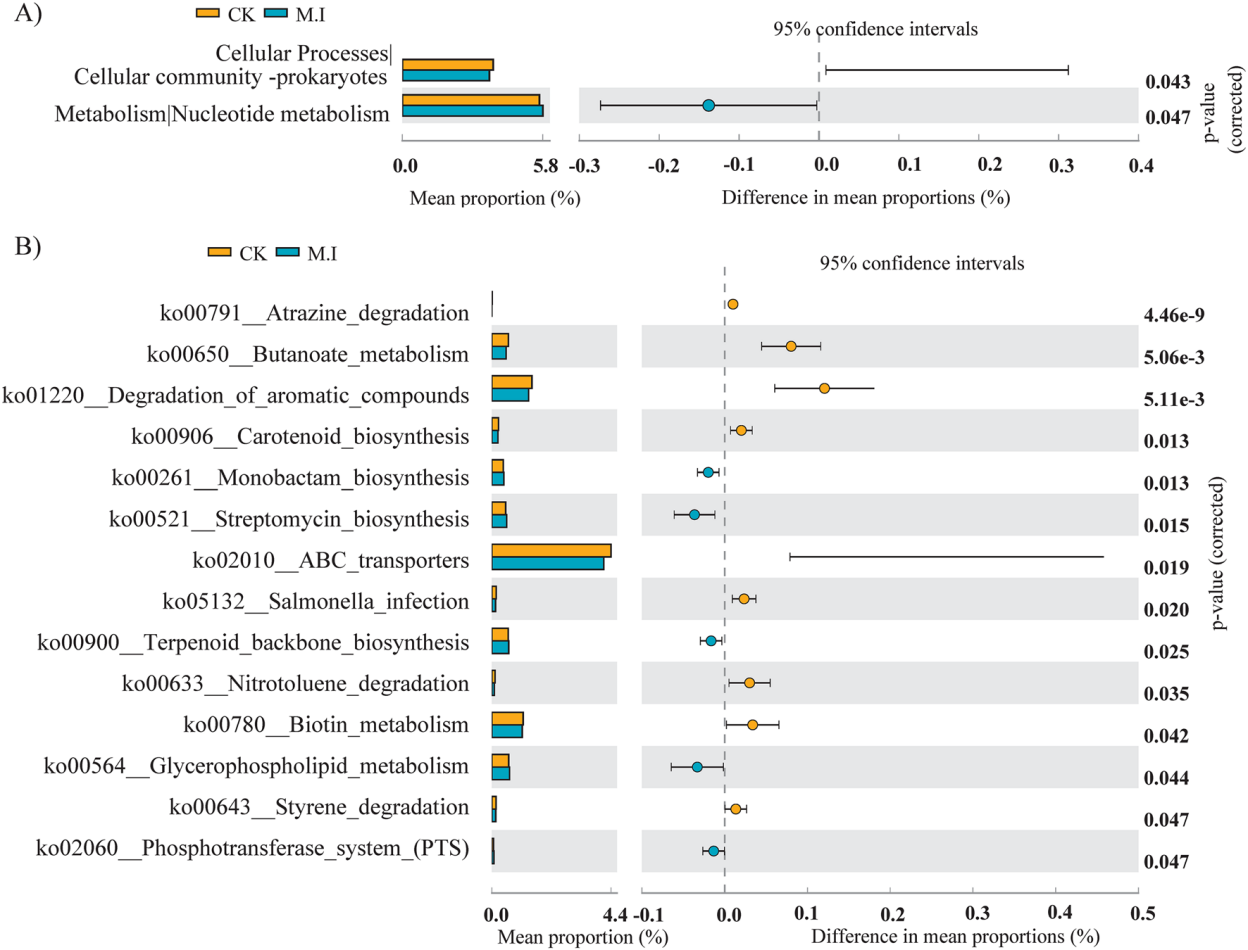
Differential microbial metabolic function of the 2nd tier (**A**) 3rd tier (**B**) KEGG in soil of the pristine forest forests and *M. micrantha*-invaded forest.

The functions of KEGG level 1 (1st tier) microorganisms were mainly divided into 6 categories, of which metabolism was the main category (ranging from 60.31 to 61.42%), followed by environmental information processing (ranging from 11.62 to 12.13%) (Fig. 3A). NMDS showed that there was significant separation between the CK forest and the forest invaded by *M. micrantha*, indicating that the invasion of *M. micrantha* significantly changed the soil microbial metabolic function (Fig. 3B). The function of KEGG level 2 (2nd tier) was significantly different between the two treatments according to the t test, and level 3 (3rd tier) metabolic functions were further analyzed for differences. As shown in Fig. 4A, we found that *M. micrantha* invasion significantly increased the metabolism of nucleotides, while the metabolism of cellular processes (cell community-prokaryotes) decreased significantly. Further analysis of significantly different metabolic functions in the 3rd tier (Fig. 4B) showed that after the invasion of *M. micrantha*, the monomer biosynthesis of monolactam and streptomycin increased (*p* < 0.05). Terpenoid backbone biosynthesis, the phosphotransferase system and glycerophospholipid metabolism (a component of the cell membrane) were also significantly increased (*p* < 0.05).

### Changes in functional gene expression of carbon, nitrogen and phosphorus cycling

**Figure 5:**
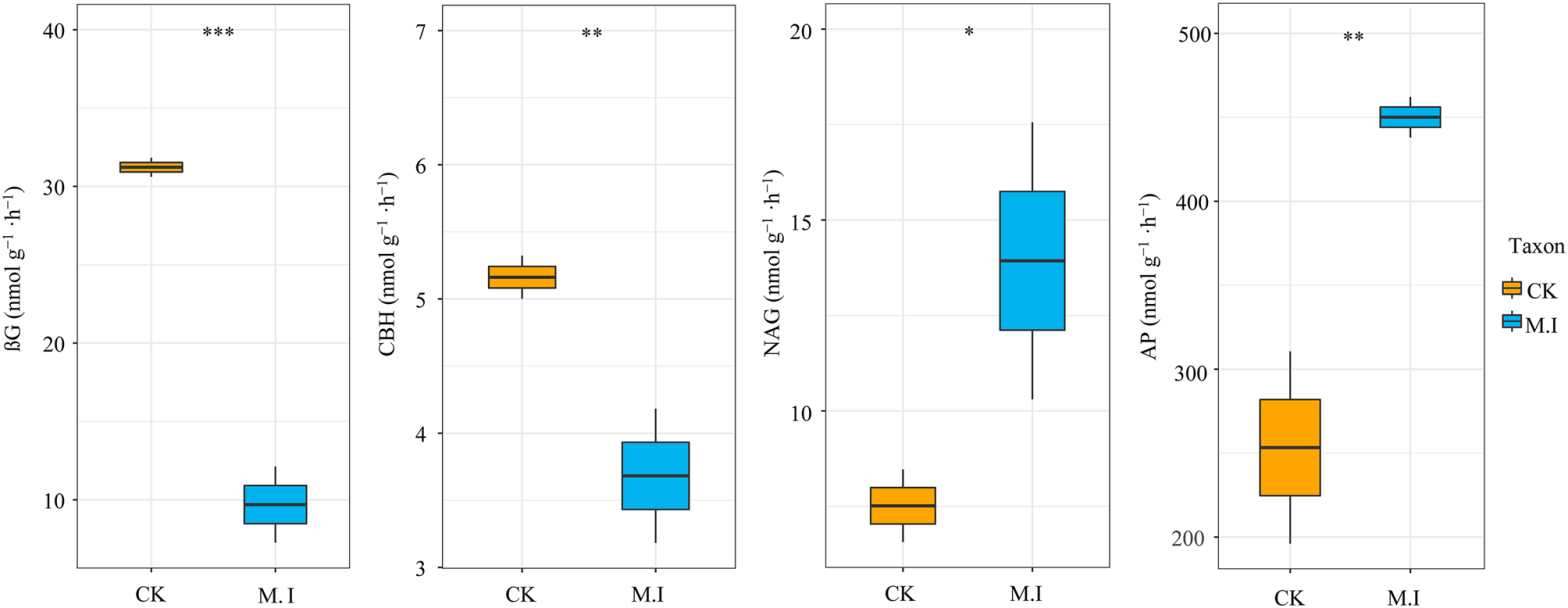
Enzyme activities in soil of *M. micrantha* invasive and non-invasive forests. βG: β-glucosidase (EC 3.2.1.21), CBH: cellobiohydrolase (EC 3.2.1.91), NAG: N-acetyl-β-D-glucosaminidase (EC 3.2.1.30), *AP* AP: Acid phosphatase (EC 3.2.3.2).

**Figure 6:**
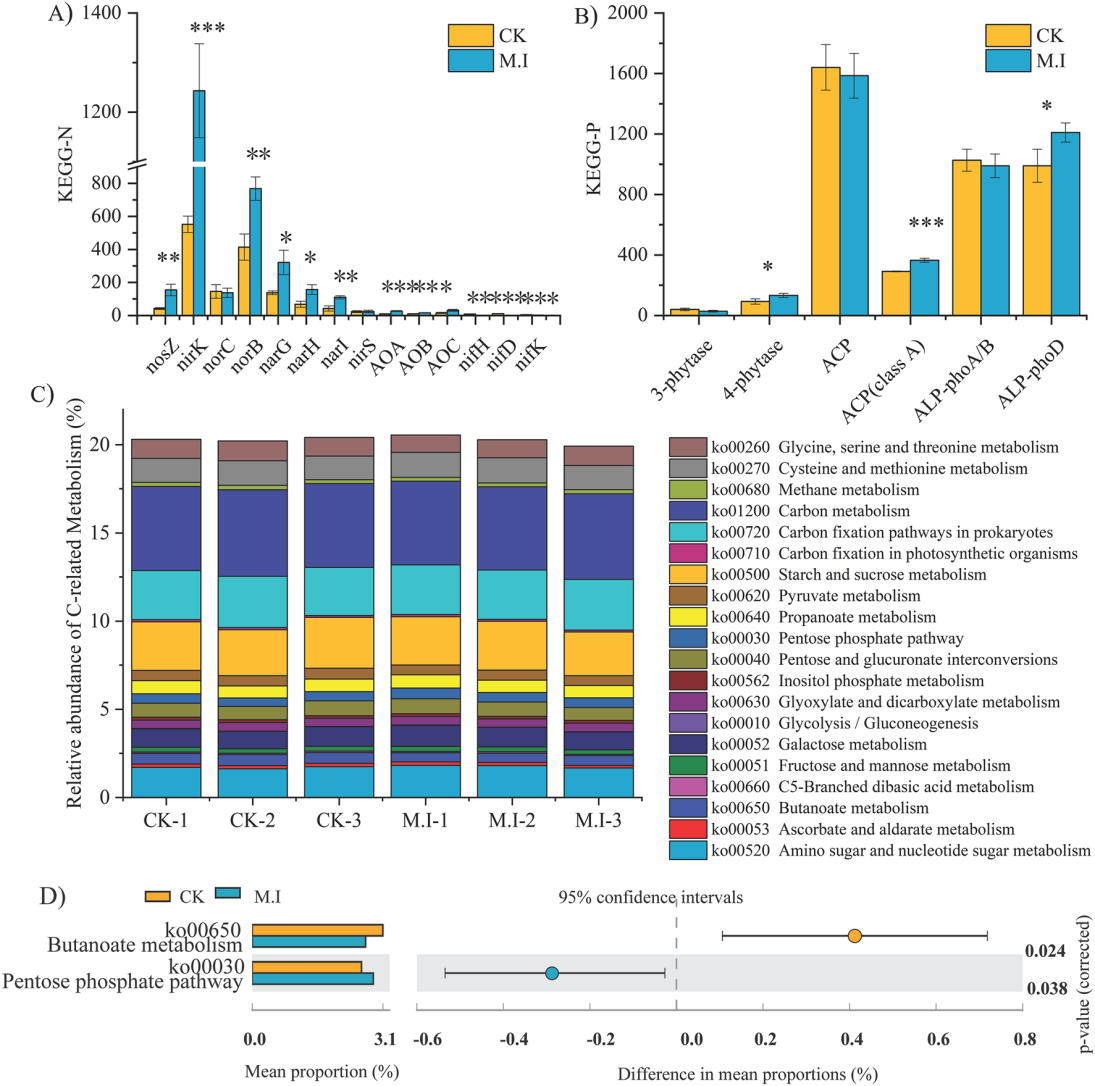
Changes of functional gene expression of carbon, nitrogen and phosphorus cycling after *M. micrantha* invasion. (**A**) The absolute abundance of genes coding for nitrogen cycling. (**B**) The absolute abundance of genes coding for phosphorus mineralization (**C**) The relative abundance of genes coding for carbon cycling. (**D**) Differential microbial functional genes coding for carbon cycling.

We found that βG and CBH decreased significantly after *M. micrantha* invasion, but NAG and AP increased significantly (Fig. 5). We also explored the microbial functions related to carbon, nitrogen and phosphorus nutrients, and the results showed that the functional genes of nitrogen and phosphorus were more easily affected by *M. micrantha* invasion than those of carbon (Fig. 6). After *M. micrantha* invasion, several nitrification bacteria (AOA, AOB and AOC) and denitrification (nosZ, nirK, norB, narG, narH and narI) and nitrogen-fixation genes (nifH, nifD and nirK) increased (*p* < 0.05) (Fig. 6A). The invasion of *M. micrantha* increased (*p* < 0.05) the activities of 4-phytase, ACP (class A) and ALP-phoD phosphorus-related enzymes (Fig. 6B). We counted 20 carbon-related metabolic function genes (Fig. 6C), and after a t test, we found that butanoate metabolism decreased and the pentose phosphate pathway increased after *M. micrantha* invasion, while other carbon metabolism pathways did not change significantly (Fig. 6D).

### Soil microbial composition; and functional gene expression of carbon, nitrogen and phosphorus cycling response to physical and chemical properties of soil

**Figure 7:**
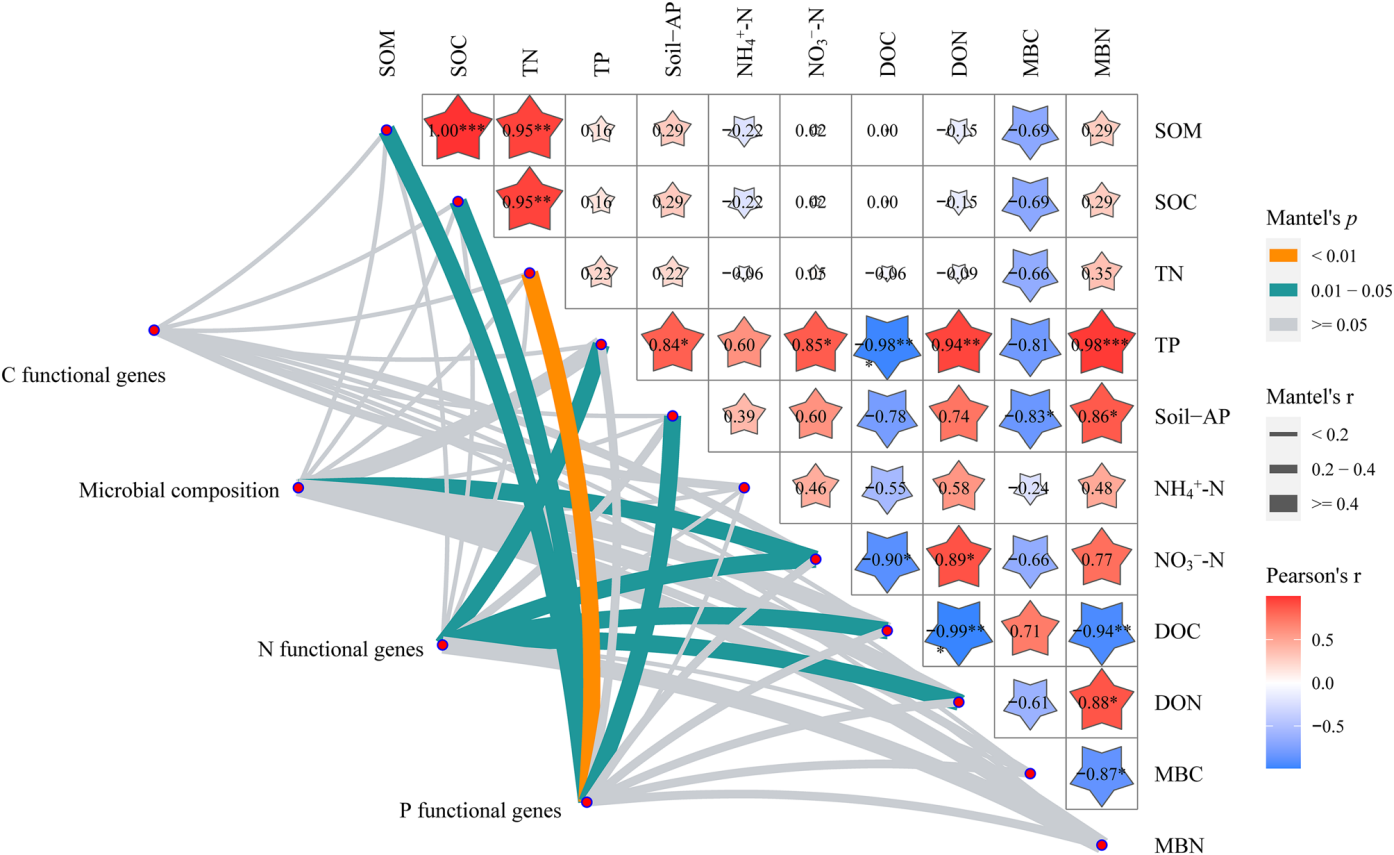
Environmental drivers of soil microbial community after *M. micrantha* invasion. Microbial community composition (based on relative abundances at the phylum level) and functional gene expression of carbon, nitrogen and phosphorus cycling (based on biochemical KEGG modules) was related to each environmental factor by partial (geographic distance–corrected) Mantel tests. Edge width corresponds to the Mantel’s r statistic for the corresponding distance correlations, and edge color denotes the statistical significance based on 9,999 permutations.

Correlation analysis showed that there was a significant correlation between soil nutrients and different microbial compositions (Fig. S1A) and functional genes (Fig. S1B). For example, there was a significant positive correlation between Proteobacteria and SOM and a significant negative correlation between Actinobacteria and MBC, TP, AP, and NO_3_^–^-N (*p* < 0.05). Gemmatimonadetes was positively correlated with TP and MBN (*p* < 0.05). Soil microbial enzymes and functional gene expression of nitrogen and phosphorus cycling were significantly correlated with MBC, AP, NH_4_^+^-N and NO_3_^–^-N. Thus, to identify soil physical and chemical property drivers in our data set, we correlated distance-corrected dissimilarities of community composition and functional genes with those of environmental factors (Fig. 7). Overall, soil microbial functional gene expression of nitrogen and phosphorus cycling was significantly affected by physical and chemical properties; however, there was no significant relationship between soil microbial carbon cycle functional gene expression and soil physical and chemical properties. NO_3_^–^-N was strongly correlated with both the microbial composition (based on relative abundances at the phylum level) and soil microbial nitrogen functional genes (*p* < 0.05). SOM, SOC, TN, and soil-AP were strongly correlated with soil microbial phosphorus functional genes (*p* < 0.05). There was a significant correlation between some nutrients, such as NO_3_^–^-N, MBC, DOC and TP (*p* < 0.05).

## Discussion

### The invasion of *M. micrantha* significantly changed the soil microbial community structure and Actinobacteria and Gemmatimonadetes abundances

The interaction between soil microorganisms and plant invasion has attracted increasing attention^47^. Previous studies have shown that exotic plant invasion not only changes the physical and chemical properties of the soil but also affects the soil microbial community. Through metagenomic analyses, our study found that the soil microbial community structure and some microbial species changed after *M. micrantha* invasion (Fig. 2B,C). In the early stage, it was proven by PLFA that *M. micrantha* invasion can change the microbial community structure^16^. We further compared the different species (the species in the top 10 most abundant taxa after *M. micrantha* invasion) and found that there were significant changes in Actinobacteria and Gemmatimonadetes abundances. Carey et al.^48^ obtained the same result with *A. triuncialis* and *E. caput-medusae* as invasive species, but the changes in Actinobacteria and Gemmatimonadetes abundances were not significant. Actinobacteria are gram-positive bacteria that tend to grow in barren environments^49^ and have genes that encode the decomposition of cellulose and hemicellulose, which play an important role in the decomposition of carbon^50^. After *M. micrantha* invasion, the relative abundance of Actinobacteria decreased significantly, which affected the decomposition of SOC. Combined with carbon-related enzyme activity, we found that the SOC and MBC contents were nonsignificantly decreased but showed a downward trend. Gemmatimonadetes are gram-negative bacteria, and gram-negative bacteria tend to grow well under substrate-rich conditions^51^. The results showed that the substrate nutrients of *M. micrantha* were better after invasion, which was consistent with the significant increase in nitrogen and phosphorus nutrients, especially the contents of TP and NO_3_^–^-N, which created an environment suitable for the growth of Gemmatimonadetes.

### Soil metabolic function shift in the microbial community after invasion of *M. micrantha*

As an indicator of microbial metabolism, soil enzymes play an important role in ecosystem nutrient cycling^52^. βG, CBH, NAG and AP are considered to be the main carbon-, nitrogen- and phosphorus-acquiring enzymes^53^ in soil. We found that NAG and AP increased significantly after *M. micrantha* invasion. This result is the same as that of Li et al.^16^. The AP enzyme and CM-cellulase hydrolase increased significantly with *M. micrantha* invasion, increasing the mineralization rate of nitrogen and phosphorus and promoting the nitrogen and phosphorus nutrient cycle^16^. In addition to enzyme activity, the KEGG database integrates a comprehensive database of genes, enzymes, compounds and metabolic network information, which provides a more comprehensive view of the functions and utilities of microbes from both high-level and genomic perspectives^54^. We showed that the metabolic function of soil microorganisms changed significantly after *M. micrantha* invasion by NMDS. Similar to the results of Li et al.^17^, using CLPP, different plant communities significantly changed the catabolic capacity of microbial communities^55^. *Mikania micrantha* invasion increased substrate utilization activity and diversity, which greatly changed the functional composition of the soil microbial communities. We found that the invasion of *M. micrantha* increased the metabolism of nucleotides, weakened the metabolism of cellular community prokaryotes, and increased the biosynthesis of monobactam and streptomycin. Yin et al.^19^ found that biocontrol bacteria such as Catenulispora accumulate in the rhizosphere of *M. micrantha*, synthesizing polyketones and antibiotics such as streptomycin to inhibit pathogenic microorganisms. We also showed that *M. micrantha* invasion can effectively control plant bacterial diseases from the point of view of functional metabolism and enhance the adaptation and invasion of *M. micrantha* to various environments to a great extent. In addition, the increase in the phosphortransfer process after *M. micrantha* invasion indicates that the bacteria absorb more carbohydrates after *M. micrantha* invasion, especially hexose, hexitol and disaccharide.

### Changes in functional gene expression of carbon, nitrogen and phosphorus cycling after the invasion of *M. micrantha*

The integration of microbial community structure and function into plant invasion research can provide a great deal of knowledge about the invasion process and the mechanism of microbial-driven nutrient cycling. After confirming that *M. micrantha* invasion alters the structure and function of microorganisms, we focused on functional genes related to carbon, nitrogen and phosphorus cycles. We found that *M. micrantha* invasion significantly increased nitrification, denitrification and nitrogen fixation genes (Fig. 6A). Yu et al.^36^ also obtained the same results, confirming that microorganisms in the rhizosphere of chamomile can increase the content of available nitrogen. The structural equation model determined that the available nitrogen was obtained mainly through AOA-mediated nitrification, which accelerated the nitrogen cycle^36^. We found that in addition to AOA, *M. micrantha* invasion also significantly changed the functional abundance of AOB and AOC nitrification genes (Fig. 6A). Nitrification is the central component of the global nitrogen cycle, which is related to nitrogen mineralization, biological fixation and loss, and the process of transforming nitrate nitrogen into NO_3_^–^-N in soil. *Mikania micrantha* preferred NO_3_^–^-N over NH_4_^+^-N, and *M. micrantha* had greater root allocation and faster growth with more NO_3_^–^-N^56^. Therefore, the increase in nitrification gene abundance significantly increased the content of NO_3_^–^-N and facilitated high interspecific competition in *M. micrantha* plants, which is one of the reasons for the rapid growth and invasion of *M. micrantha*.

In addition to nitrogen, other studies have shown that improving the availability of soil phosphorus is also one of the main factors for the success of plant invasion^19^. Phosphorus is a necessary nutrient element for plant growth^29^. Yin et al.^19^ compared the rhizosphere microbial community between *M. micrantha* invasion and two native plants (*Polygonum Polygonum* and *Paeonia lactiflora*). The results showed that the enrichment of the phosphorus-solubilizing bacteria Pseudomonas and Enterobacter was helpful in increasing the available phosphorus in *M. micrantha* rhizosphere soil. The change in functional genes of the microbial community after *M. micrantha* invasion may be greater than that of species. We found that *M. micrantha* invasion increased phosphorus-related enzyme activity genes, such as 4-phytase, ACP (class A) and ALP-phoD, and the soil-AP content increased by 72.46% (Fig. 6B). In addition to microbial species, it was also confirmed that *M. micrantha* invasion could increase soil-AP content via functional metabolism.

However, *M. micrantha* invasion had no significant effect on SOM, and previous studies did not come to a consistent conclusion that soil carbon storage increased^8,9,33–35^, decreased^57–59^ or remained unchanged^60^ after *M. micrantha* invasion. Our analysis of carbon-related metabolic genes showed that butyric acid metabolism decreased and pentose phosphate metabolism increased after *M. micrantha* invasion, while most other carbon metabolism pathways had no significant changes. Ni et al.^61^ studied the relationship between carbon storage and the abundance of total PLFA microbial biomass. The results showed that the increase or decrease in soil carbon storage caused by invasion depended on microbial biomass, which had a positive impact on the growth of invasive plants^15,62–64^. Our research showed that there was no significant change in MBC after *M. micrantha* invasion, and the carbon dynamic changes caused by *M. micrantha* were more dependent on the changes in carbon-related metabolic genes. Although we found differences in microbial community structure and carbon-, nitrogen- and phosphorus-related metabolic function genes, further experiments are needed to study the proteins and metabolites^65^ of carbon and nutrient metabolism pathways.

### Differences in microbial composition and metabolic function genes after invasion of *M. micrantha* and their relationships to soil properties

The invasion of exotic plants is one of the most common threats to natural ecosystems^19^. Plants invade the ecosystem to produce more litter, which can cause changes in soil conditions, such as pH value, SWC or soil nutrients^66–70^. Correlation analysis showed that there was a significant relationship between soil characteristics and different soil microbial compositions and metabolic functions (Figs. 7 and S1). Most studies believe that the main environmental factor driving the change in soil microbial community structure is soil pH^71,72^. In our study, NO_3_^–^-N was strongly correlated with the microbial composition. Nitrogen is an essential element for all living organisms^73^ and is involved in the formation of amino acids, nucleotides, metabolic enzymes (including nitrate reductase and the membrane lipid peroxidase defense system) and other compounds necessary for life^74^ due to its participation in the synthesis of amino acids, nucleotides, and other compounds essential to life. Previous studies have shown that *M. micrantha* has a relatively high nitrification rate, significantly increasing the available NH_4_^+^-N and NO_3_^–^-N contents^75^, and *M. micrantha* prefers NO_3_^–^-N to NH_4_^+^-N. After *M. micrantha* invasion, nitrification genes (AOA, AOB and AOC; Fig. 6A) and nitrogen-fixation genes (nifH, nifD, nirK) of soil microorganisms were significantly increased, resulting in more NO_3_^–^-N. This may explain the strong correlation between NO_3_^–^-N content and microbial community composition after *M. micrantha* invasion.

Some studies also believe that SOC and AP may be the key environmental factors affecting changes in soil microbial function^16^. In this study, it was found that SOC, AP and TN were significantly correlated with soil phosphorus functional genes (Fig. 7). TP, NO_3_^–^-N, DOC and DON were significantly correlated with soil nitrogen functional gene (Fig. 7). In tropical forest ecosystems, there is highly weathered soil and recalcitrant P fraction formation, soil erosion, and leaching losses^76,77^. Phosphorus has become an important limiting factor in primary production and other ecological processes. For example, phosphorus plays an important role in many metabolic processes, including signal transduction, energy transfer, respiration, macromolecular biosynthesis^78^ and nitrogen fixation^79^. Under the condition of phosphorus limitation, *M. micrantha* invasion increased the contents of AP and TP in soil (not significantly, which may be related to season), thus affecting the functional genes of soil microbial nitrogen and phosphorus. DOC and DON are the most important components of dissolved organic matter (DOM). DOM is the most active and cycling organic component and plays an important role in affecting the dynamics and interaction of nutrients and microbial functions, thereby serving as a sensitive indicator of shifts in ecological processes^80^. Some studies have shown that there is a significant positive correlation between DON content and NO_3_^–^-N content. A large amount of NO_3_^–^-N is easily reduced to NO_2_^–^, and reacts with SOM to form DON^81,82^. This is consistent with our results. As DOM is easily used by microorganisms, DOC and DON promote the growth of microorganisms and affect nitrogen functional genes.

## Conclusions

Our study highlighted the changes in the composition and function of the microbial community after *M. micrantha*, such as the increase in the relative abundance of Gemmatimonadetes and the increase in streptomycin biosynthesis metabolism. *Mikania micrantha* significantly affects the functional metabolism of microbial nitrogen and phosphorus by mobilizing the activities of microbial enzymes related to carbon, nitrogen and phosphorus in soil and increasing nitrogen and phosphorus nutrients and DOM in soil, thus forming a positive soil-microbial relationship, which is beneficial to the growth of *M. micrantha*. Taken together, these results indicate that combining plant eco-physiology, microbial composition, and metabolic and soil geochemistry can provide a better mechanistic understanding and prediction of invasion feedback processes.

## Supplementary Information

Supplementary Figure S1.

## Data Availability

All data supporting the findings of this study are available in the paper or from the corresponding author upon request.
