# Supplementary material for: Significant changes in soil microbial community structure and metabolic function after Mikania micrantha invasion
Source: Sci Rep. 2023 Jan 20;13:1141. doi: 10.1038/s41598-023-27851-6 (PMC9860029; doi:10.1038/s41598-023-27851-6)

Fig.S1 Correlation between microbial composition (A), metabolic function (B) and soil biochemical properties in soil of *M. micrantha* invasive and non-invasive eucalyptus forests.


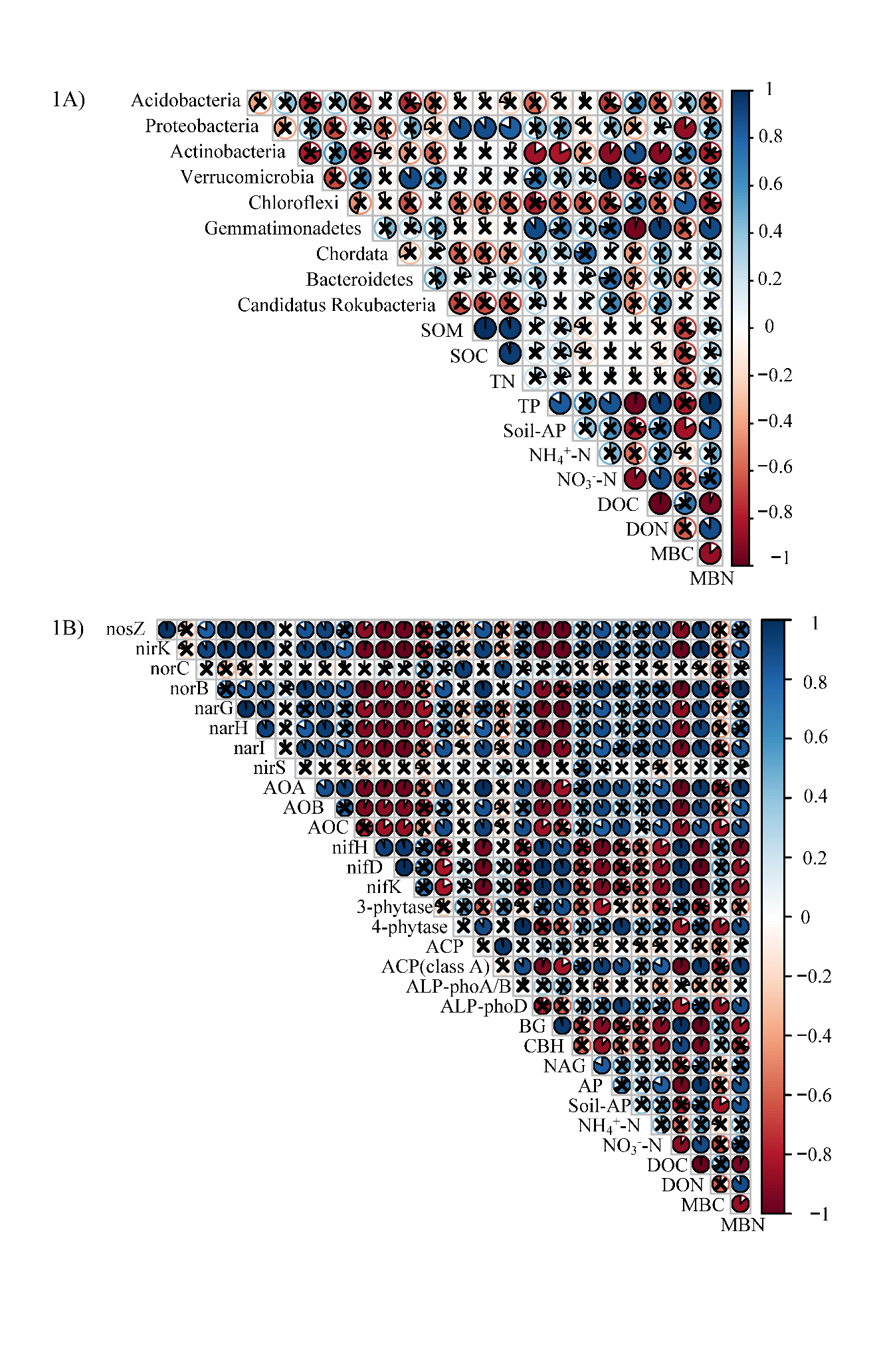

Supplement: Supplementary file 1 — Supplementary Figure S1. [file 41598_2023_27851_MOESM1_ESM.docx]
